# Dr. Reynell Coates’ Contributions to Surgery

**Published:** 1842-07-16

**Authors:** Reynell Coates


MEDICAL EXAMINER.
NEW SERIES.
No. 29.] PHILADELPHIA, JULY 16, 1842. [Vol. I.

### *Contributions to Surgery.*

BY REYNELL COATES, M. D.

*Note on the means of avoiding Excoriations of the Heel and Perineum in Fractures of the Lower Extremities, treated by Permanent Extension.*

Concluded from p. 337.

The requisition for variety of matter in the pages of a weekly Journal renders it proper to close this somewhat protracted note with a few practical remarks, already pledged in the preceding paragraphs.

I have spoken of the fact that the fractured limb is occasionally found to be of the full length when the surgeon is first called to the case. This condition of things, though most common in severe injuries producing great prostration or collapse, or in patients who are in a state of extreme debility at the time of the accident, is by no means confined to such. It is not very uncommon for the fragments to escape all longitudinal displacement for some hours after the fracture, even in athletic subjects who have been carefully handled and undressed without awkwardness. For this reason an accurate admeasurement and comparison between the relative length of the two limbs (from the anterior superior spinous process of the ossa ilia to the apices of the external malleoli,) should be made prior to the application of the apparatus, or, at least, before any attempt at extension; for it would be folly to subject the heel and perineum to the undue action of extending forces where simple retention is alone requisite. With a proper examination every forty-eight hours, when an inextensible apparatus is employed, such cases may be occasionally conducted to a happy termination without disturbing a single band during the entire course of treatment.

The surgeon is often called to the charge of neglected or maltreated cases after the lapse of two, three, or four weeks from the time of fracture. Such cases, which are invariably attended with shortening of the limb, and often with other deformities, will be found in very various degrees of advancement towards a permanent union, according to the temperament and health of the patient, and the extent to which the fragments have been allowed to remain displaced. Even under very unpromising circumstances, we should not hastily abandon attempts at extension, in these instances, from dread of excoriation, nor suffer ourselves to be deterred from diminishing inevitable evils because certain experimenters have ventured to dictate to nature how long a time she shall be allowed for effecting the several stages of union,—the glutinous, the condroid, the provisional and the permanent bony union. From several dissections in subjects dying from other diseases during the process of fractures, I am convinced that as long as tolerably free angular motion between the fragments can be produced by slight forces, the reticulated fibres of the temporary osseous deposits do not extend from one portion of the injured bone to the other, and as long as this is the case, the chondroid connections are capable of yielding gradually, but extensively, to moderate extension, such as may be well borne without danger of excoriation. They may even do so at a later period, when the limb supports its own weight in the horizontal position without the occurrence of angular deformity, though at this time it is reasonable to conclude that the reticulated bony fibres traverse the entire callus in considerable numbers, forming solid bridges uniting the fragments. That newly formed bone may be made to yield to strong and lasting pressure, as the comparatively delicate structure of the young bones will do, is sufficiently proved by the occasional occurrence of shortened or deformed limbs in patients who have been discharged from the care of the surgeon, and have pursued their usual avocations for some weeks before the disastrous consequences of too early exercise force themselves upon their attention. Cases of this character are likely to become more frequent hereafter, if the prevalent love of surgical display should lead to a continuance of the unwise disposition recently betrayed by some operators,—that of hurrying their fracture cases out of the hospitals, and publishing reports of them at the earliest possible moment. The case of an adult, who has been treated for a fracture of the thigh or leg, discharged and made the subject of a report in three, four, or five weeks from the time of the accident, is only calculated to mislead us in endeavouring to estimate the usual time required for the formation of an available temporary or *provisional* union. An examination of such cases after the lapse of a month from the time of the report, would probably present us with many patients walking upon limbs materially shortened, and distorted with angular deformities from strong muscular action and the weight of the body, although the maltreated subjects may have trodden with tolerably firm steps upon beautifully formed limbs at the time of the discharge. Except in fractures occurring so near the large joints as to cause a dread of limited motion from rest too long continued, and when passive motions of the limb appear insufficient to remove the danger, it should never be considered legitimate in surgery to allow the patient, if an adult, to leave his bed until the completion of the fourth week, nor should he be permitted to test his ability to support the weight of the body upon the injured limb, or to walk without the aid of a crutch, until the completion of full six weeks, although many patients might literally take up their beds and walk a long distance at an earlier period, and many days might elapse before the evil consequences of their undue haste would become apparent. In constitutions broken down by intemperance, and in patients of phlegmatic temperament, the time required for an available temporary union may be protracted to two or even three months, though there may be no tendency to an arrest of the process endangering the formation of pseudarthrosis. Persons of this character are not unfrequently discharged after the longest ordinary term of confinement, walking with great facility, and with limbs free from all deformity ; yet, after a few weeks the injured members become bowed, and sometimes shortened, so as to render them—it may be, not always justly—permanent advertisements of the disgrace of the surgeon. I have dwelt the more strongly upon this subject, which is merely collateral with our argument, because a number of cases of fracture reported as cured in astonishingly short periods of time, have appeared in the Journals recently, unaccompanied by the cautions demanded to prevent these instances of seemingly brilliant success from proving too seductive to those who are not very familiar with such accidents. It should be borne in mind that, though we may *retard a union* by bad management in diet or manipulation, we cannot by any means *sensibly accelerate it ;* so that in the cases to which reference is made, no part of the credit of the rapid cure can be reflected upon the mode of treatment and the surgeon. If, in those cases, no evil followed the early discharge, most assuredly they were happy exceptions to general rules, and not legitimate incentives to imitation in other places and upon other subjects. If the awfully severe, though wonderfully successful dissections of our distinguished countryman, Dr. Parker of Canton—before some of which our " Waterloo operations" hide their diminished heads—had been performed on men of European or American mould, many of them must have proved inevitably mortal.

But—to return from this digression—as long as motion can be produced in the seat of fracture, we need not despair of diminishing displacements resulting from neglect or malpractice by mechanical means. Even long after all motion ceases it is possible to remove considerable angular deformities by a well regulated apparatus, as I have had recent occasion to observe in several instances. Firm and frequently repeated extension *by the hands of the surgeon*, coupled with a proper apparatus for permanent retention, will accomplish great good sometimes in cases of malposition remaining several weeks after fracture; and not only may this be effected without producing excoriation, but even when ulceration already exists it may be cured under the action of the apparatus, proving how greatly exaggerated is the common idea of the force required for effective extension and counter-extension, when rightly applied.

The following outline of a case illustrating many points touched upon in the present paper is quoted from memory, the original notes being now at a distance. It has been frequently narrated in my lectures, and may have appeared in some former publication more in detail.

About the year 1825, a young stout man, by profession a butcher, fell and fractured the shaft of the femur. His habits had been vilely intemperate, and a temperament originally sanguine had lapsed into the phlegmatic. This being the first case of the kind occurring in the practice of a young surgeon, since then an extensive practitioner, he employed the apparatus of Dr. Hartshorne with the stuffed sock and screw, so strongly condemned in the early part of this note. The plan adopted was that of exceedingly gradual extension, his preceptor, in office consultation, recommending him to aim at bringing the limb to the full length in about two weeks. The heat of the sock, and the pressure of the extending band, rendered the patient extremely restive, and he made almost daily complaint of the pain of extension. By the advice of the preceptor, who did not see the case, the extending band was relaxed for a few hours, whenever it was complained of, and again tightened at the surgeon's next visit. This, as usual, led to continually increasing intolerance of pressure, and the attempts at extension were rendered almost nugatory in their effect. About the conclusion of the third week, the surgeon discovering that great shortening still existed, became alarmed for his reputation, and requested me to share the responsibility of the case. On examination I found the limb shortened—as nearly as my memory serves—about three inches and a half. There was extensive superficial excoriation and some marked ulceration of the perineum, occasioned by the counter-extending pad of this apparatus ; and on the back of the heel was a very deep gangrenous patch, covering the tendo Achilles, caused by the sock and extending band: this gangrene was not circumscribed. The chondroid union between the fragments was pretty far advanced, but yielded to powerful extension by means of the hand to the extent perhaps of half an inch. Pronouncing the impossibility of perfect reduction without refracturing the bone, (which could not have been well effected until the union became much firmer and more osseous,) I held the case by no means hopeless if vigorously treated.

The apparatus was condemned, the limb suffered to lie undressed upon the bed, and the excoriations and gangrenous spot were repeatedly bathed with brandy. It was agreed that the apparatus of Dr. Physick should be employed from the moment that a line of demarcation began to appear around the gangrenous part. This occurred, I think, on the third day ; and extension and counter-extension, on the principles laid down in this note, were immediately instituted. By very frequent visits and careful efforts, the limb was brought down at least two inches in two or three days, the bands acting directly over the diseased surfaces, which were protected by a simple dressing, and this renewed as seldom as possible. Something continued to be gained very slowly by the extension from day to day for perhaps a week, and fortunately the constitutional condition of the patient rendered the progress of reunion unusually slow. Under the pressure of the bands, the ulceration and excoriation of the perineum cicatrised completely, while the slough over the heel rapidly separated deeply around the edges. After the final removal of the apparatus, the patient traversed his chamber for some time by the aid of his crutch ; and I think about the end of the second month he returned to his usual avocations, *and among them his intemperance.* His limb was then shortened perhaps an inch, for I cannot recall the exact measurement, but he did not require a high heeled shoe, nor was his slight limp important to one in his situation in life. The slough of the heel involved part of the thickness of the tendo Achilles, and this portion—the rest having been clipped away with scissors—did not separate for some time after his return to business ; the ulcer requiring to be dressed for weeks. Yet, although no change took place in the relations of the fragments during the long interval in which this slough compelled him to visit his surgeon frequently, I met him many months afterwards walking with a very unsightly angular deformity of the limb, consequent upon a slow yielding of the new bone under the pressure of the body and the exercise of heavy lifting. This certainly did not begin to appear till at least four months after the accident, and was not due to reabsorption of the callus ; for the union had become perfectly firm, and the deformity had ceased to increase. The debility consequent upon a long confinement, and the soreness of the heel, had probably prevented those exercises which occasioned the angular displacement until after he had ceased to visit his surgeon.

